# Real-world risk stratification for coronary heart disease: a one-year prediction model using health information exchange data

**DOI:** 10.1186/s12889-025-24266-y

**Published:** 2025-09-30

**Authors:** Yaqi Zhang, Yifu Mo, Naoto Ozawa, Takumi Ichikawa, Chao-Jung Huang, Zhi Han, Lu Tian, Shaun T. Alfreds, Karl G. Sylvester, Doff B. McElhinney, Xuefeng B. Ling

**Affiliations:** 1 College of Automation, Guangdong Polytechnic Normal University, Guangzhou, China; 2 School of Medicine, Stanford University, Stanford, CA USA; 3 China Southern Power Grid Company Limited, Guangzhou, China; 4 Nippon Life Insurance Company, Osaka, Japan; 5 Joint Research Center for Artificial Intelligence Technology and All Vista Healthcare, National Taiwan University, Taipei, Taiwan; 6 HealthInfoNet, Portland, ME USA

**Keywords:** Coronary heart disease, Predictive algorithm, Multiple multivariate machine learning, Risk stratification, Electronic health records

## Abstract

**Background:**

Coronary heart disease (CHD), the most common form of heart disease, progresses over years before culminating in serious cardiac events. Early prediction and intervention are critical to reducing CHD-related morbidity, mortality, and healthcare burden.

**Objective:**

To develop and validate a machine learning model using statewide electronic health records (EHRs) to predict 1-year risk of CHD in the general population of Maine, enabling targeted preventive strategies.

**Methods:**

Two population-based cohorts were constructed from the Maine Health Information Exchange (HIE): a retrospective cohort for model training and calibration (2015–2017, *N* = 1,042,124), and a prospective cohort for external validation (2016–2018, *N* = 1,040,158). EHR features included demographics, diagnoses, procedures, medications, labs, and utilization metrics. A multistage modeling pipeline—comprising statistical filtering, XGBoost-based feature selection, risk prediction, and isotonic regression calibration—was used to construct the final model. Validation included discrimination, calibration, and survival analysis.

**Results:**

The final XGBoost model achieved strong discrimination: AUC = 0.952 (95% CI: 0.950–0.954) in the retrospective cohort and 0.888 (95% CI: 0.885–0.890) in the prospective cohort. Based on calibrated risk probabilities, the population was stratified into five risk categories: very low (92.30%, *N* = 960,021), low (6.79%, *N* = 70,676), medium (0.85%, *N* = 8,888), high (0.05%, *N* = 554), and very high (0.002%, *N* = 19). Among the very high-risk group, 11 individuals (57.89%) developed CHD within one year.

**Conclusions:**

This statewide, HIE-based CHD risk prediction model demonstrates robust performance and real-world applicability. It enables early identification of high-risk individuals and supports population-scale precision prevention through evidence-informed, proactive care.

**Supplementary Information:**

The online version contains supplementary material available at 10.1186/s12889-025-24266-y.

## Introduction

Coronary heart disease (CHD)—also known as coronary artery disease or ischemic heart disease—is a leading cause of morbidity and mortality globally, affecting 6.7% of U.S. adults aged 20 years and older [1]. In 2013, CHD ranked among the ten most expensive conditions treated in U.S. hospitals, with estimated inpatient costs exceeding $9 billion [2], and its medical expenditures are projected to nearly double between 2015 and 2030 [3]. Given its burden, timely risk stratification and early intervention are essential to reducing preventable complications, long-term disability, and healthcare costs [4].

Traditional clinical scoring systems such as the Framingham Risk Score (FRS) [5] and the ACC/AHA 2013 Risk Score [6] have provided a foundation for CHD risk estimation. However, these tools exhibit well-documented Limitations. The FRS, originally designed for 10-year risk estimation, performs suboptimally for shorter time frames (< 5 years) and fails to account for dynamic changes in risk factors. It also overemphasizes age, underestimates lifetime risk in women, and lacks generalizability due to its derivation from a predominantly White population. These limitations compromise its utility in contemporary, diverse, and dynamic care settings.

A 1-year prediction horizon offers a critical intermediate window—longer than emergency risk scores (e.g., 30 days), but more actionable than 10-year estimations. This window aligns with the typical biological progression of vulnerable atherosclerotic plaques, which often rupture within 12 months of becoming unstable [7, 8]. Early identification of individuals at elevated 1-year CHD risk enables clinicians to initiate intensive preventive strategies—such as lipid-lowering, anti-inflammatory therapy, and lifestyle modifications—before clinical events occur [9].

Over the past two decades, more than 45 CHD prediction models have been developed and compared, with most achieving moderate discriminatory accuracy (AUC 0.65–0.85) [10]. However, these models frequently rely on limited sample sizes, static variables, and traditional statistical methods. Many lack scalability and fail to integrate the full complexity of longitudinal clinical data captured in real-world settings.

Machine learning (ML) algorithms provide a data-driven alternative, capable of modeling complex, non-linear relationships in high-dimensional datasets. ML methods can automatically extract predictive features from sparse and correlated clinical data, making them well-suited to the challenges of risk prediction in electronic health records (EHRs) [11, 12]. The increasing adoption of EHRs across hospitals, clinics, laboratories, and pharmacies [13] offers unprecedented opportunities to use routinely collected data for real-time risk assessment and decision support [13].

Health Information Exchanges (HIEs) aggregate patient-level EHR data across diverse care settings and providers. This infrastructure enables population-scale risk stratification and supports value-based care through timely, predictive analytics. ML models integrated with HIE platforms have been applied to diverse conditions—including hypertension [14], lung cancer [15], chronic kidney disease [16], and arrhythmias [17]—but predictive modeling for CHD using statewide, HIE-connected EHR data remains underexplored.

In this study, we developed and validated a machine learning-based CHD risk pre-judgement model using a statewide HIE-EHR dataset from the State of Maine. Our goal was to predict individuals’ risk of developing CHD within the subsequent year. By identifying high-risk patients early, our model enables timely clinical intervention and facilitates precision prevention strategies at both individual and population levels—ultimately improving outcomes and optimizing healthcare resource allocation.

## Method

### Ethics statement

This study was conducted in accordance with the principles of the Declaration of Helsinki. The raw dataset—covering approximately 95% of the population of Maine—was obtained from HealthInfoNet, the state’s Health Information Exchange. All protected health information was de-identified prior to analysis. As the study involved only de-identified data and did not include human subjects research as defined by federal regulations, it was determined to be exempt from review by the Stanford University Institutional Review Board.

### Experimental design and workflow

**Fig. 1:**
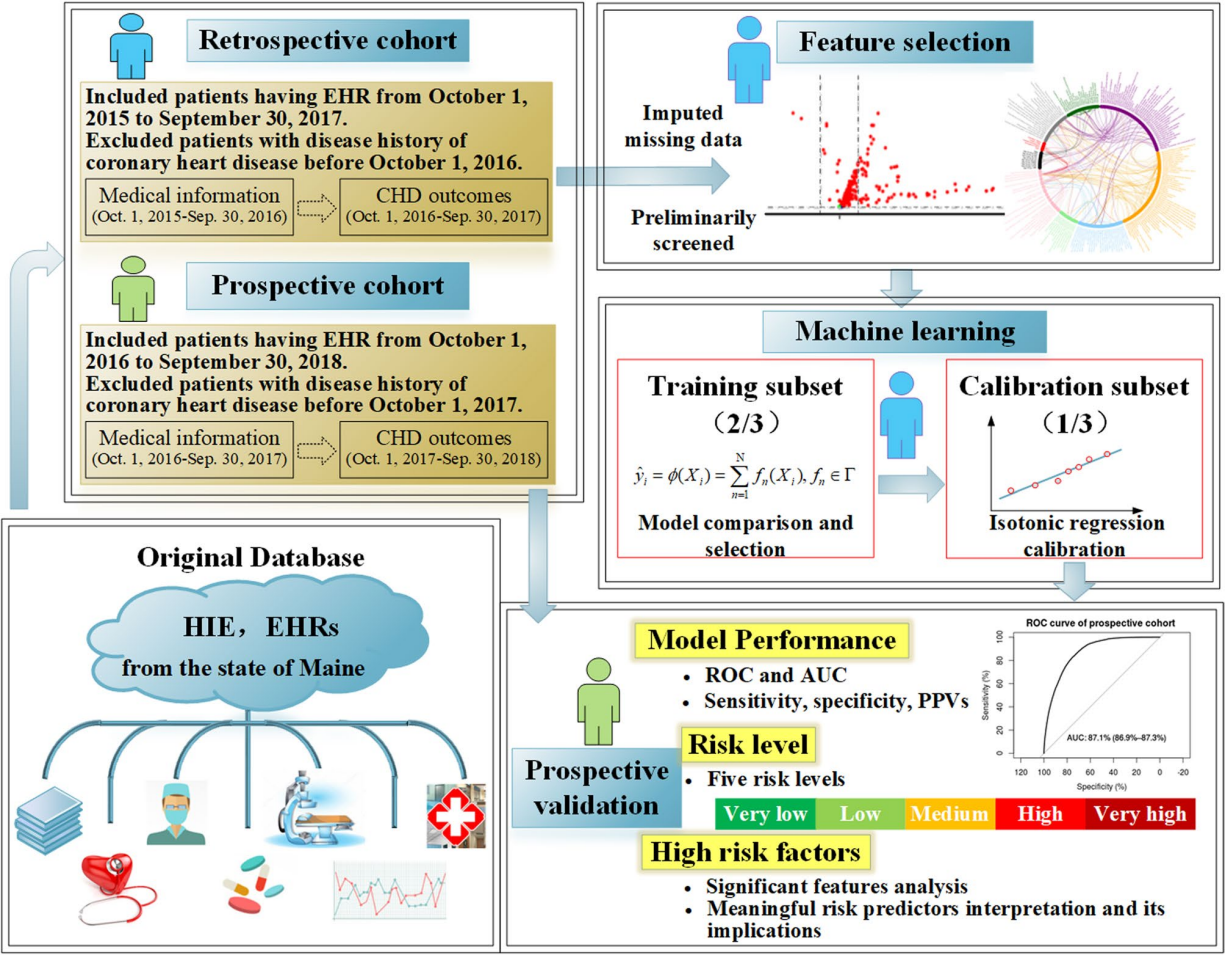
The schematic study workflow

Figure 1 depicts a schematic workflow of the study, including cohort enrollment, inclusion/exclusion criteria, modeling process, and application.

### Dataset

Our predictive algorithm was developed to estimate the risk of incident CHD over a one-year horizon, using clinical data from the preceding year obtained through Maine’s statewide HIE, HealthInfoNet. The dataset captured patient encounters across 35 hospitals and 34 federally qualified health centers in Maine between October 1, 2015, and September 30, 2018.

CHD was defined according to ICD-10-CM (International Classification of Diseases, 10th Revision, Clinical Modification) codes and included: (1) obstructive coronary artery disease characterized by narrowed or blocked vessels; (2) non-obstructive coronary artery disease presenting as angina without significant vessel obstruction; and (3) spontaneous coronary artery dissection, a frequently underrecognized cause of myocardial infarction.

The retrospective cohort utilized EHRs from October 1, 2015, to September 30, 2017. Predictor variables were derived from data spanning October 1, 2015, to September 30, 2016, while the outcome window extended from October 1, 2016, to September 30, 2017. After excluding individuals with a documented CHD diagnosis during the baseline Year, the final retrospective cohort included 1,042,124 patients, of whom 15,881 developed incident CHD during the follow-up period.

The prospective cohort consisted of individuals with EHR data available from October 1, 2016, to September 30, 2018. Model inputs were drawn from the period October 1, 2016, to September 30, 2017, and incident CHD outcomes were assessed from October 1, 2017, to September 30, 2018. After applying the same exclusion criteria for pre-existing CHD, the prospective cohort comprised 1,040,158 patients, among whom 15,768 developed CHD within the subsequent year.

In both cohorts, exclusion of individuals with a prior CHD diagnosis ensured that the model was trained and validated exclusively on incident CHD events.

### Feature selection

To improve computational efficiency and model relevance, feature selection began with a targeted literature review to identify variables previously associated with CHD. These candidate risk factors included demographic characteristics, laboratory test results, chronic medical conditions, and prescription medication histories. Laboratory values were classified as “normal” or “abnormal” based on facility-specific thresholds defined by participating institutions in the HIE. Chronic conditions were captured using primary and secondary ICD-10-CM diagnosis codes, while medication exposure was quantified as the frequency of prescriptions for a given drug over the past year.

In the final dataset, laboratory results were encoded as binary indicators: 0 for “normal” and 1 for “abnormal.” Missing lab values were imputed using a clinically informed strategy based on the premise that unperformed tests typically reflect clinician judgment of low risk, rather than data loss. Accordingly, missing results were imputed as “normal” (0). This approach aligns with real-world clinical decision-making and was validated through sensitivity analyses. A detailed explanation of the imputation strategy is provided in *Additional file 1: Model technical aspects*.

To reduce dimensionality and minimize noise, a chi-square test was applied as an initial feature pre-screening step to eliminate variables with no significant univariate association with CHD incidence. This statistical filtering narrowed the feature set to 1,928 binary and categorical variables. The rationale and implementation of chi-square screening are also described in *Additional file 1: Model technical aspects*.

Subsequently, the XGBoost algorithm was applied to the filtered feature set. Features with positive importance scores were retained, yielding 387 variables that contributed meaningfully to predictive performance. To control for false positives, multiple hypothesis testing correction was performed, and the retained features met a predefined false discovery rate (FDR) threshold [18, 19].

### Model building and evaluation

The retrospective cohort was randomly partitioned into two subsets: one for model training and the other for model calibration, the latter used to transform raw prediction scores into calibrated risk probabilities.

During the training phase, we evaluated a range of machine learning algorithms, including Least Absolute Shrinkage and Selection Operator (LASSO) [20], Feed-forward Neural Network (FNN) [21], Random Forest (RF) [22], Boosting [23], Extreme Gradient Boosting (XGBoost) [24], Naïve Bayes (NB) [25], and k-Nearest Neighbor (KNN) [26]. To enhance predictive performance and robustness, we also implemented a Bayesian probabilistic ensemble framework [27, 28], which integrates outputs from multiple base learners through weighted aggregation and consensus voting. This ensemble approach leverages the strengths of diverse algorithms to improve overall model accuracy and generalizability.

**Fig. 2:**
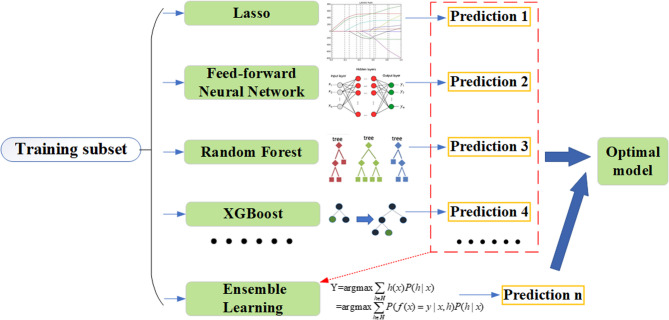
The modelling process with multi-algorithms

The complete modeling pipeline—including training, feature selection, algorithm comparison, and ensemble prediction—is illustrated in Fig. 2.

During model development, three key hyperparameters were optimized: maximum tree depth, number of boosting rounds (weak learners), and learning rate. The search space was defined as follows: maximum tree depth (3–10), number of weak classifiers (100–300), and learning rate (0.05–0.2). Grid search combined with K-fold cross-validation was employed to identify the optimal hyperparameter combination. Special attention was given to mitigating overfitting from overly deep trees, ensuring a sufficient number of learners to capture data complexity, and balancing learning rate to optimize convergence speed and model stability. The final model was trained using 70% of the retrospective cohort, with performance evaluated on the remaining 30% to assess generalizability.

For calibration, we applied isotonic regression, a nonparametric technique well-suited for capturing non-linear distortions in predicted probabilities. Using the calibration subset from the retrospective cohort, we mapped the model’s raw prediction scores (ŷ) to empirical positive predictive values (PPVs)—defined as the proportion of individuals with observed CHD events among those with predicted scores ≥ ŷ. This calibration process yielded clinically meaningful risk scores expressed as PPVs, which were then applied to the independent prospective cohort.

Each individual in the prospective cohort was assigned a calibrated CHD risk score, and subsequently stratified into five ordinal risk categories: very low, low, medium, high, and very high, based on increasing predicted risk thresholds. Risk scores in this study represent the probability of incident CHD events within one year and are thus directly interpretable as PPVs.

To evaluate the model’s discriminatory ability, we computed the receiver operating characteristic (ROC) curve and the area under the curve (AUC) for both retrospective and prospective cohorts. Calibration curves comparing observed versus predicted risk were generated across the full spectrum of scores and across clinically meaningful subpopulations. Model performance was further summarized using threshold-based metrics, including sensitivity, specificity, PPV, NPV, positive likelihood ratio, and negative likelihood ratio.

For all performance metrics, 95% confidence intervals (CIs) were calculated using bootstrap resampling (*n* = 100 iterations). Bootstrapping was chosen for its robustness to the non-normality and skewness typical of real-world EHR data. Each iteration resampled the original dataset with replacement to generate variability estimates and construct CIs.

Finally, to evaluate time-to-event risk stratification, we conducted multivariable Cox proportional hazards regression within the prospective cohort. Kaplan–Meier survival curves were plotted for each risk group to visualize differences in cumulative CHD incidence over time. All analyses were performed using R statistical software.

## Results

### Population baseline characteristics

Table 1 summarizes the baseline characteristics of the retrospective and prospective cohorts. Demographic and clinical features were similarly distributed between the two groups, supporting the comparability of both populations. All disease classifications were coded using ICD-10-CM standards. In particular, the category of cardiovascular diseases (CVD) included diagnoses such as heart failure, rheumatic mitral valve disease, atrioventricular and left bundle branch block, cardiomyopathy, nonrheumatic aortic, tricuspid, and mitral valve disorders, atherosclerosis, and other disorders of arteries and arterioles.

**Table 1:** Baseline features of the two cohorts (retrospective and prospective)

Characteristics	Retrospective (*N* = 1,042,124)	*n* (%)	Prospective (*N* = 1,040,158)	*n* (%)
Age(years)				
< 19	210,710	20.22	215,840	20.75
19–34	183,836	17.64	186,584	17.94
35–49	180,117	17.28	178,566	17.17
50–64	244,853	23.50	240,283	23.10
65–74	131,435	12.61	132,263	12.72
75–84	63,168	6.06	61,148	5.88
> 85	28,005	2.69	25,474	2.45
Gender				
Male	462,859	44.41	461,006	44.32
Female	579,265	55.59	579,152	55.68
Chronic disease				
CVD	45,118	4.33	58,654	5.64
Chronic obstructive pulmonary disease	28,711	2.76	38,390	3.69
Occlusion and stenosis of precerebral artery, not result in cerebral infarction	4,544	0.44	6,701	0.64
Chronic kidney disease	16,408	1.57	21,228	2.04
Angina pectoris	1,151	0.11	1,620	0.16
Other peripheral vascular diseases	7,804	0.75	11,853	1.14
Overweight and obesity	52,265	5.02	85,694	8.24
Hypothyroidism	60,610	5.82	74,878	7.20
Acute disease				
Acute myocardial infarction	1,424	0.14	1,821	0.18
Pain in throat and chest	45,428	4.36	52,346	5.03
Abnormalities of breathing	40,593	3.90	46,075	4.43
Other soft tissue diseases	75,736	7.27	82,254	7.91
Abnormal findings on diagnostic imaging of lung	10,928	1.05	12,909	1.24
Disease events				
Essential (primary) hypertension	66,174	6.35	56,678	5.45
Type 2 diabetes mellitus without complications	37,592	3.61	29,296	2.82
Chronic obstructive pulmonary disease	10,391	1.00	10,682	1.03
Hyperlipidemia	1,293	0.12	647	0.06
Health status				
Presence of cardiac and vascular implants and grafts	5,913	0.57	6,010	0.58
Tobacco use	26,739	2.57	19,356	1.86
Body mass index > 30	48,258	4.63	59,753	5.74
Personal history of certain other diseases	34,201	3.28	39,993	3.84
Encounter for other aftercare and medical care	18,443	1.77	16,139	1.55
Lab test				
Erythrocyte distribution width RBC	81,910	7.86	61,238	5.89
Platelets in Blood	98,218	9.42	73,342	7.05
Basophils in Blood	83,948	8.06	63,453	6.10
Cholesterol in high-density lipoprotein	78,829	7.56	29,979	2.88
Anion gap in Serum or Plasma	87,597	8.41	58,451	5.62
Hemoglobin A1c/Hemoglobin.total in Blood	35,692	3.42	19,086	1.83
Glomerular filtration rate	21,139	2.03	17,453	1.68
Magnesium in Serum or Plasma	24,035	2.31	21,903	2.11
Medication				
Nitrate Vasodilator	6,518	0.63	4,197	0.40
Loop Diuretic	21,703	2.08	18,799	1.81
P2Y12 Platelet Inhibitor	4,070	0.39	2,980	0.29
Dihydropyridine Calcium Channel Blocker	27,453	2.63	27,093	2.60
Biguanide	29,351	2.82	29,234	2.81
Insulin Analog	14,493	1.39	13,795	1.33
Angiotensin 2 Receptor Blocker	25,671	2.46	26,001	2.50
Alpha-Adrenergic Blocker	23,903	2.29	22,996	2.21

### Model performance

**Fig. 3:**
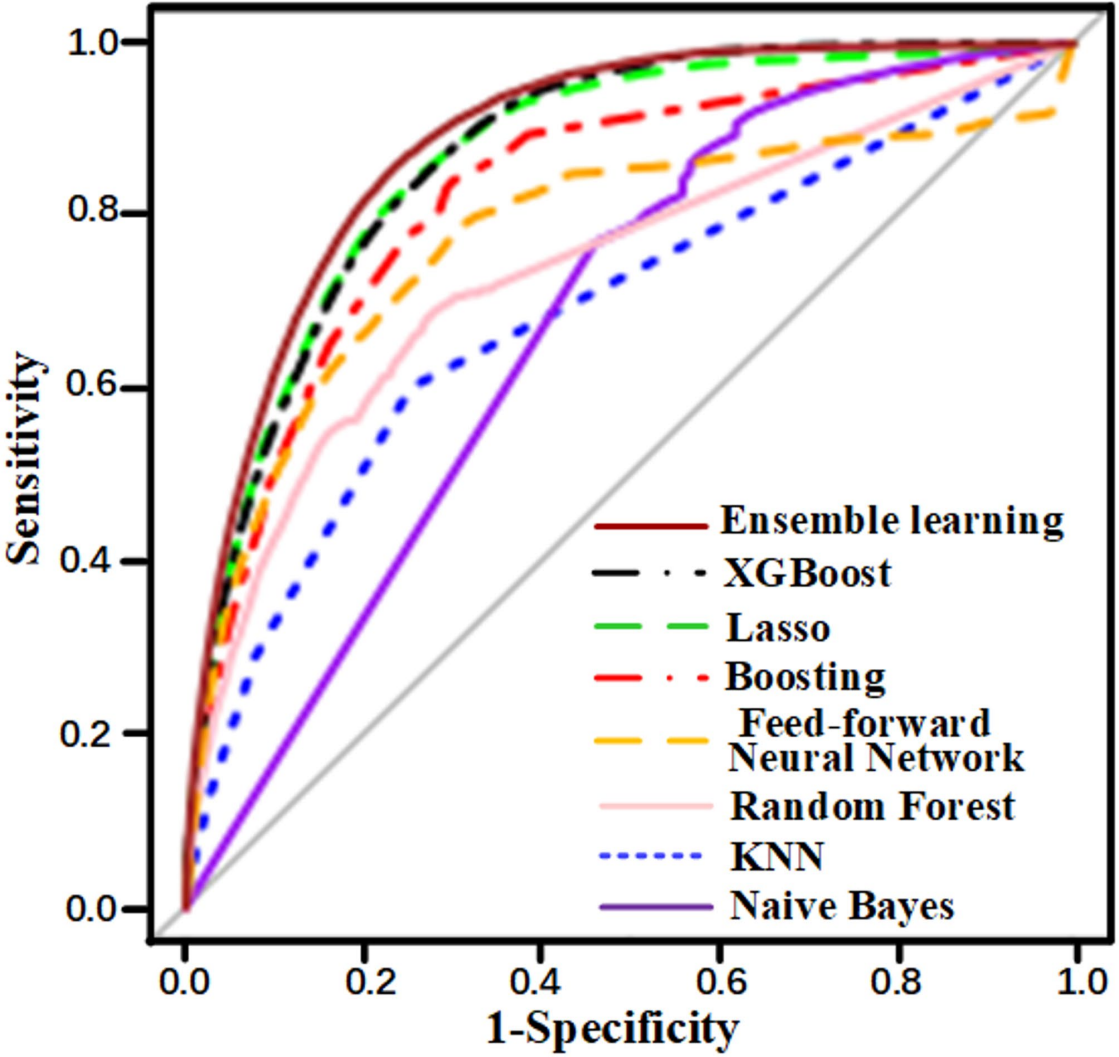
The ROC comparisons of the model performance in the prospective cohort

As shown in Fig. 3 and Supplementary Fig. 1, the ensemble learning method achieved the highest overall performance, with an AUC of 0.952 (95% CI: 0.950–0.954) in the retrospective cohort and 0.888 (95% CI: 0.885–0.890) in the prospective cohort. The XGBoost model, selected as the final predictive algorithm, achieved a prospective AUC of 0.871 (95% CI: 0.869–0.873) (Supplementary Fig. 2). Notably, LASSO also demonstrated comparable performance to XGBoost but lacked several practical and modeling advantages.

While LASSO offers simplicity and performs well in high-dimensional settings through regularization-based feature selection, XGBoost provides several key benefits. It effectively models non-linear relationships and feature interactions, which are prevalent in complex EHR data. XGBoost also handles missing values natively and is more robust to outliers, improving generalizability in real-world datasets. Moreover, it supports individual-level interpretability through SHAP (Shapley Additive exPlanations), facilitating clinical transparency and explainability.

Given its predictive accuracy, robustness to clinical data variability, scalability, and interpretability, XGBoost was selected as the final model for deployment. The model’s calibration performance, validated using isotonic regression and visualized with 95% confidence intervals, is shown in Supplementary Fig. 3.

### Feature community structure and correlation networks

The final XGBoost model identified 229 features with non-zero predictive importance for 1-year CHD risk. These features spanned key domains of patient health, including: demographics (*n* = 2), chronic diseases (*n* = 48), acute conditions (*n* = 51), disease-related events (*n* = 46), health status indicators (*n* = 21), laboratory test results (*n* = 20), medication usage (*n* = 20), medical procedures (*n* = 16), and healthcare utilization metrics (*n* = 5), such as emergency visits, outpatient visits, and total medical costs from the preceding Year. A detailed breakdown of feature types and distributions is provided in Supplementary Table 1.

Among the top 80 most influential features, several disease diagnoses were associated with markedly elevated risk. Conditions such as heart failure, angina pectoris, acute myocardial infarction, cardiomyopathy, occlusion and stenosis of the precerebral artery, atherosclerosis, hypertensive heart disease, and nonrheumatic aortic valve disorders all demonstrated odds ratios (ORs) exceeding 7.0, highlighting their strong associations with future CHD events. Additionally, several medication classes emerged as key predictors, including nitrate vasodilators, loop diuretics, P2Y12 platelet inhibitors, dihydropyridine calcium channel blockers, biguanides, and insulin analogs. Laboratory tests such as erythrocyte distribution width, platelet count, basophil count, and HDL cholesterol were also significant contributors. Measures of healthcare utilization—particularly emergency visits, outpatient encounters, and annual medical costs—further enhanced the model’s ability to identify high-risk individuals.

**Fig. 4:**
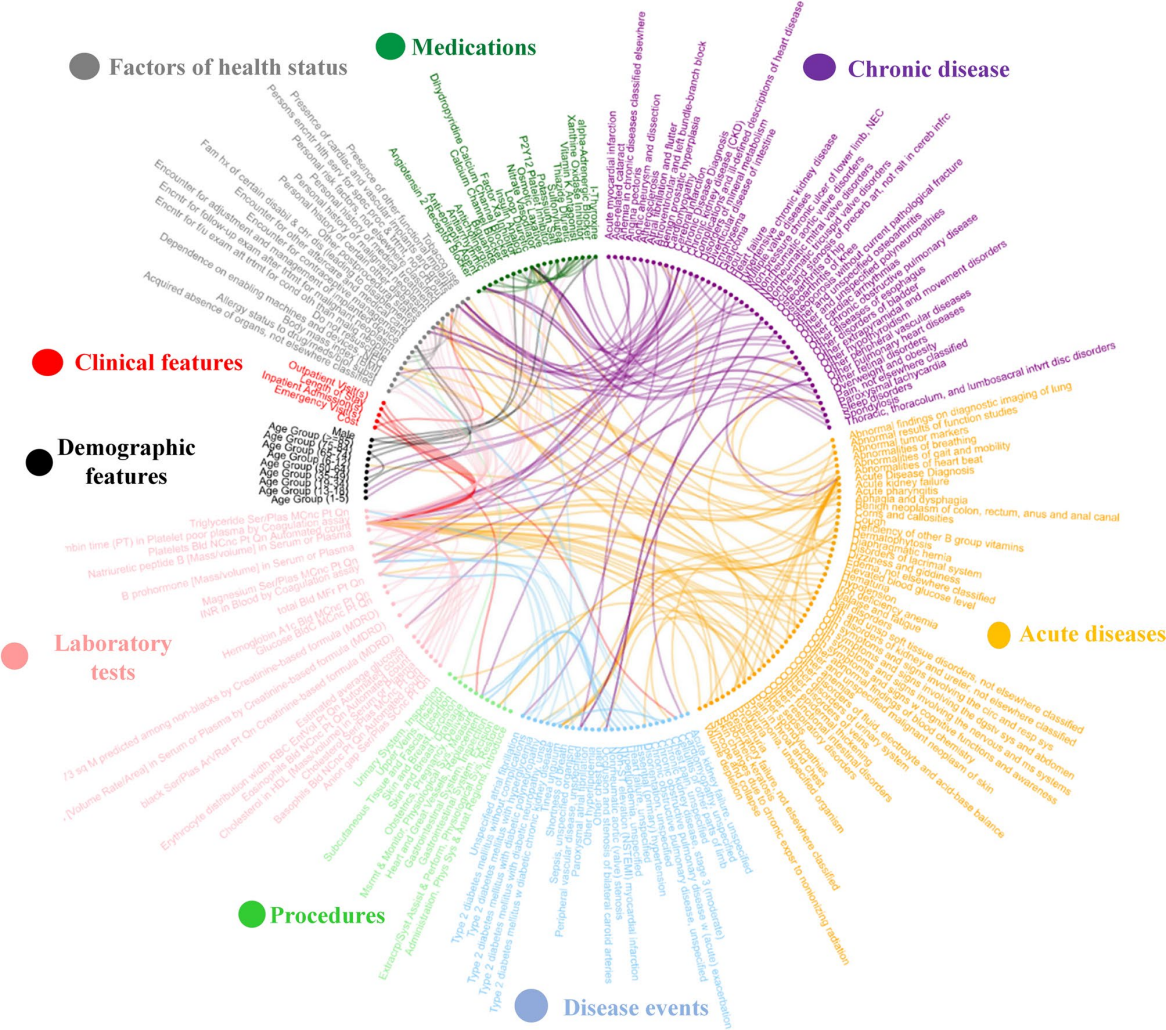
The community structure containing 193 impactful features. Each line represents one correlation that was significant (the correlation coefficient > 0.1 or < −0.1)

To explore inter-feature dependencies, we constructed a Spearman correlation network using the selected features. The network (Fig. 4) included 212 nodes (features) and 204 edges (correlations >|0.1|). Diagnostic features (*n* = 103) and demographic variables (*n* = 11) formed the densest subnetwork, but meaningful correlations were also observed among medications (43 edges), lab test abnormalities (82 edges), and health status indicators (48 edges). While procedural (12 edges) and utilization-related features (8 edges) exhibited fewer direct correlations, they remained connected within the broader network. These findings underscore the complex and interconnected nature of EHR-derived predictors, supporting the need for multivariate, interaction-aware modeling approaches such as XGBoost.

### Event pre-judgement of population at high risk for CHD in the future one year

**Fig. 5:**
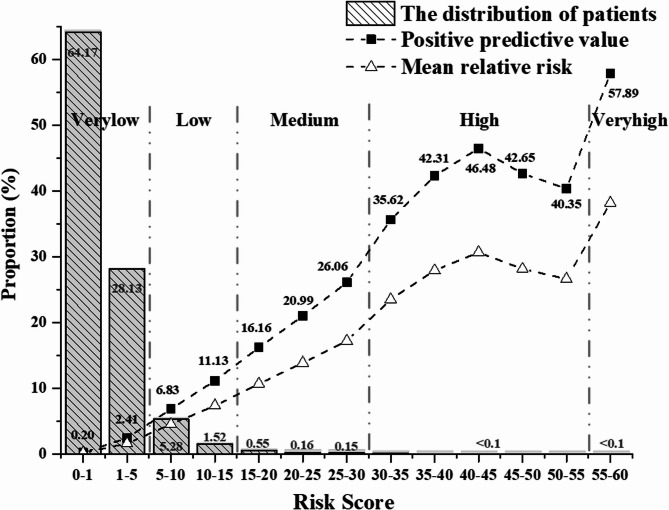
The distributions of patients, PPVs and mean relative risk with different risk scores in the prospective cohort

As shown in Fig. 5, our calibrated risk prediction model stratified individuals in the prospective cohort into five ordinal risk groups for future 1-year CHD events: very low, low, medium, high, and very high. The distribution and performance metrics for each group—including positive predictive value (PPV), sensitivity, and mean relative risk—are detailed in Supplementary Table 2.

The stratification results demonstrated a pronounced skew toward the lower risk categories. Specifically, over 99% of individuals (1,030,697 out of 1,040,158) were classified into the very low or low-risk groups. In contrast, only 0.06% of patients (*n* = 573) were categorized into the high and very high-risk strata.

Against a baseline 1-year CHD incidence rate of 1.6% in the full cohort, the stratified results revealed a clear gradient in event rates across the risk spectrum. The very low-risk group had an observed 1-year incidence of 0.87% (8,367 of 960,021), while incidence rose progressively across the low (7.79%), medium (18.77%), high (38.99%), and very high (57.89%) groups. These gradients affirm the model’s ability to distinguish increasing levels of absolute risk.

**Fig. 6:**
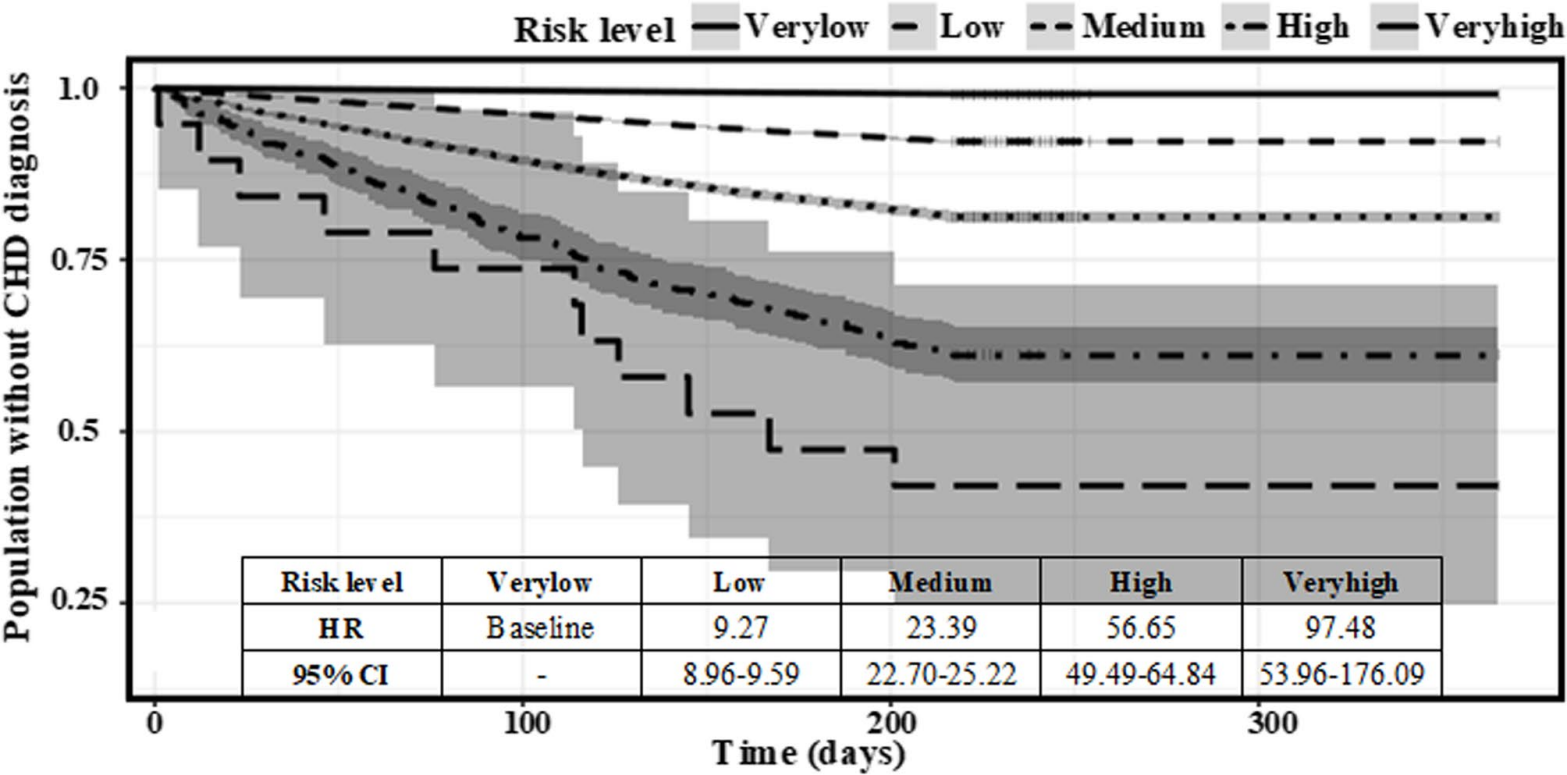
Time to the events of the confirmative CHD diagnoses: five risk subgroups in the prospective cohort. HR: hazard ratio

To evaluate time-to-event differentiation across strata, Kaplan–Meier survival curves were plotted by risk group and are presented in Fig. 6. The curves showed significant divergence, with estimated hazard ratios (HRs) ranging from 9.27 to 97.48, validating the model’s capacity to predict not just binary outcomes but also risk dynamics over the 12-month follow-up period. These results demonstrate that the model can robustly stratify the general population of Maine into clinically meaningful risk tiers, supporting proactive CHD prevention strategies at both the individual and population levels.

### Significant features

To enhance interpretability and support clinical translation, the distributions of key predictive features across the five CHD risk categories are presented in Table 2. This allows for visualization of how feature prevalence changes with increasing predicted risk, thereby offering insight into the clinical profile of each stratum.

Additionally, to explain individual feature contributions to the model’s predictions, we applied TreeSHAP (SHapley Additive exPlanations for tree-based models). The SHAP summary plot, shown in Supplementary Fig. 4, highlights the most influential features and their directional impact on predicted CHD risk. This provides transparent, model-agnostic interpretability and facilitates clinician-facing explanations at both the population and patient levels.

**Table 2:**
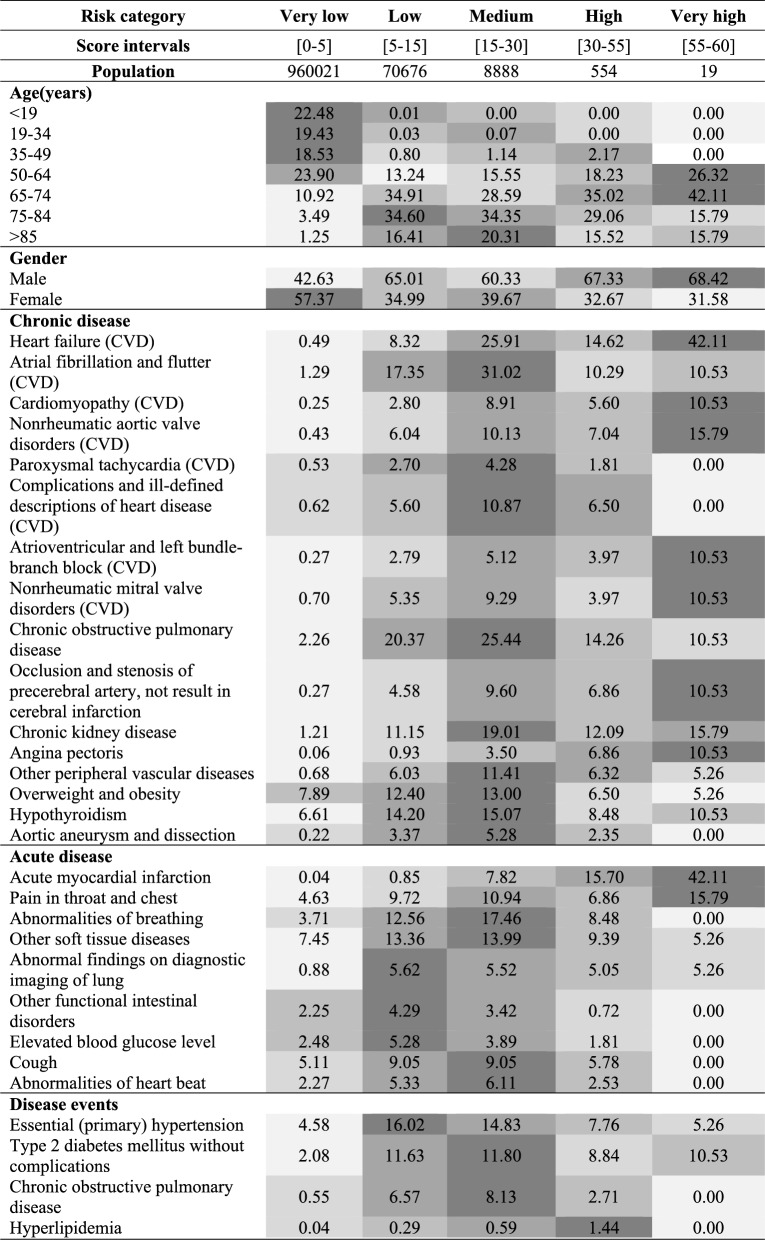

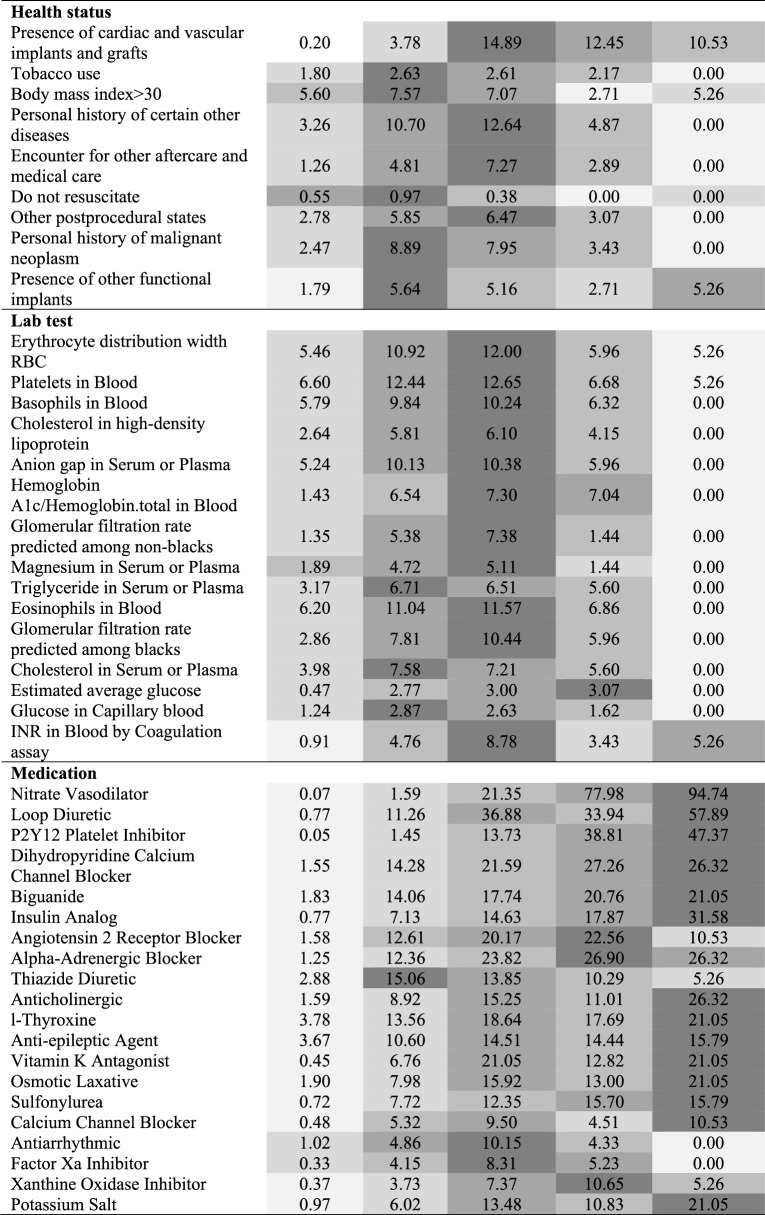
The top risk predictors distributions across the five risk categories in the prospective cohort. Thevalue represents the actual proportion (%) of people in each predicted risk population who have acertain predictor. The depth of the background color indicates the size of the proportion

As illustrated in Table 2 and Supplementary Fig. 5, the distribution of age and sex varied significantly across the five CHD risk groups. Younger individuals (under 35 years) were concentrated in the very low-risk group, comprising over 41% of that category. In contrast, individuals aged 50 Years and older constituted nearly 100% of the low, medium, high, and very high-risk groups. Additionally, males were more prevalent than females in the medium, high, and very high-risk strata, reflecting known sex-based disparities in CHD risk.

Patients classified into high and very high-risk groups also demonstrated a substantially greater burden of comorbid conditions, as shown in Table 2 and Supplementary Fig. 5. These included chronic cardiovascular diseases (e.g., heart failure, cardiomyopathy, aortic valve disorders) and acute events (e.g., myocardial infarction, chest pain). Notably, Supplementary Fig. 6 presents distinct time-to-event trajectories among chronic disease subgroups stratified by risk. For example, individuals with angina pectoris in the high-risk group exhibited a lower short-term CHD incidence than those with other chronic cardiovascular conditions, underscoring the heterogeneity within risk strata.

Table 2 further summarizes the distribution of health status indicators, laboratory test abnormalities, and medication prescriptions across risk levels. A progressive increase in the proportion of patients with abnormal values was observed as predicted risk increased. For instance, 32% of individuals in the high and very high-risk groups exhibited at least one abnormal health status factor, compared to 12.6% in the very low and low-risk groups. Similar trends were evident for abnormal lab results and the use of cardiovascular or renal-related medications.

These findings are reinforced by the TreeSHAP analysis (Supplementary Fig. 4), which confirms that many of the same clinical features contributed strongly to individual risk predictions. Collectively, these results highlight the biological plausibility and clinical face validity of the model’s risk stratification logic, underscoring its potential value for real-world implementation in precision prevention workflows.

## Discussion

This study presents a novel, EHR-based risk pre-judgement model for predicting one-year CHD events in the general population using statewide HIE data from Maine. Our model achieved strong discrimination with an AUC of 0.952 (retrospective cohort) and 0.888 (prospective validation), outperforming prior models that typically report AUCs between 0.71 and 0.81 [29–31]. These gains are attributable to two main factors: (1) the inclusion of the entire statewide population, reducing selection bias and increasing generalizability; and (2) the integration of high-dimensional EHR data across demographic, clinical, laboratory, and utilization domains, which enabled a more holistic and timely risk assessment.

### Model comparison and final algorithm selection

Among several machine learning methods evaluated, ensemble learning achieved the highest overall AUC, while XGBoost and LASSO showed comparable performance. XGBoost was selected as the final model due to its superior ability to capture non-linear interactions, robust handling of missing data, and compatibility with SHAP-based interpretability. In contrast, neural networks and random forest models underperformed—likely due to the sparsity and tabular structure of the EHR data, which favors models like XGBoost and LASSO that are less prone to overfitting under high-dimensional constraints. These observations are consistent with known bias-variance tradeoffs and are further detailed in our model performance rationale.

### Clinical risk features and interpretation

Risk stratification revealed a strong, biologically plausible gradient: individuals in higher risk groups were older, more likely to be male, and had a significantly higher burden of chronic and acute cardiovascular conditions (Table 2, Supplementary Table 1). Predictors such as heart failure, myocardial infarction, angina pectoris, atherosclerosis, cardiomyopathy, diabetes, and hyperlipidemia were highly enriched in the high- and very-high-risk groups—aligning with well-established CHD pathophysiology [31–36]. Elevated triglycerides, low HDL cholesterol, abnormal erythrocyte distribution width, and insulin use further emphasized the metabolic and inflammatory underpinnings of CHD [37]. Tobacco use, which synergizes with hypertension, dyslipidemia, and diabetes to amplify CHD risk [38, 39], also emerged as a relevant predictor. These findings reinforce the clinical validity of the model.

### Model calibration and time-to-event evaluation

To improve clinical interpretability, predicted scores were calibrated using isotonic regression and mapped to positive predictive values (PPVs). Patients were stratified into five risk groups. Incidence increased from 0.87% in the very low-risk group to 57.89% in the very high-risk group. Kaplan–Meier curves demonstrated clear time-to-event divergence (Fig. 6), with hazard ratios ranging from 9.27 to 97.48. Notably, 31.57% of patients in the very-high-risk group developed CHD within four months. This confirms that the model not only identifies elevated 1-year risk but also prioritizes individuals with imminent risk—critical for time-sensitive intervention.

### Comparative value over framingham risk score

While the Framingham Risk Score (FRS) remains a valuable tool for long-term cardiovascular risk, our model offers a complementary, more dynamic approach for near-term (1-year) prediction. Unlike the FRS, which relies on a static set of variables and was built on a predominantly White cohort, our model integrates a wider spectrum of clinical features—including updated labs, medication usage, comorbidities, and healthcare utilization patterns. This allows more personalized, population-specific predictions and addresses known FRS limitations in underestimating risk in women and younger adults. The one-year window also aligns with the average timeline for plaque destabilization and rupture, offering a critical opportunity for timely preventive action.

### Sources of uncertainty and model refinement strategies

Variability in predictions—particularly in high-risk strata—may stem from multiple sources: (1) inherent noise or missingness in EHR data; (2) clinical heterogeneity among high-risk individuals; (3) small sample size in the very-high-risk group (*n* = 19); and (4) incomplete capture of CHD etiologic complexity. To address this, future model refinements will explore enriched data inputs (e.g., social determinants, biomarkers), non-linear ensemble architectures, and subgroup interaction modeling. Bootstrapped confidence intervals and calibration plots (Supplementary Fig. 3) were added to enhance interpretability and quantify uncertainty.

### Limitations

Several limitations warrant consideration. First, socioeconomic, lifestyle, and genomic variables were not included due to data availability constraints. While contextual SDOH (Social determinants of health) data (e.g., neighborhood deprivation) may help approximate these effects, individual-level integration remains an unmet need across most U.S. HIEs. Second, the use of real-world data introduces noise and variability, though we mitigated this via code aggregation and pre-processing. Third, generalizability may be limited by the geographic focus on Maine, despite broad state-level coverage. Finally, the very-high-risk group was small; however, its strong predictive PPV reinforces the utility of even rare-risk categories for targeted prevention. 

### Implementation strategy and clinical integration

Translation into practice requires thoughtful integration. Working with the Maine HIE, we embedded the model into a population health dashboard that delivers risk scores to clinicians and accountable care organizations (ACOs) in real time. These scores can trigger alerts for preventive evaluation during primary care or wellness visits. To support uptake, we developed educational modules, visual risk flags, and SHAP-based explanations for provider-facing interpretability. Our implementation framework emphasizes seamless clinical workflow alignment, data security, and bias audits to ensure equitable deployment. Early pilot results suggest high engagement, with feedback informing refinements to threshold selection and decision support.

### Path toward generalizability and national impact

To expand scalability, we are collaborating with HIEs across multiple states for external validation and performance benchmarking. These efforts include stratified analyses by race, socioeconomic status, and geographic setting, along with assessments of clinical intervention impact. Our goal is to refine the model for national deployment, enabling its use across diverse patient populations and healthcare systems. These results will be reported in future publications.

## Conclusions

This study presents a novel, HIE-integrated risk pre-judgement tool for predicting one-year coronary heart disease risk across the general population of Maine. Unlike prior models based on limited or disease-specific cohorts, our approach leverages a comprehensive, statewide EHR dataset, enhancing generalizability and enabling population-scale risk stratification. By operating directly within the HIE infrastructure, the model delivers real-time risk scores without requiring additional input from providers—facilitating efficient, large-scale implementation.

The model holds broad applicability across stakeholders in the healthcare ecosystem:

- Clinicians can receive embedded risk assessments within EHR systems to inform proactive, personalized prevention strategies.
- Health systems and payors can use the tool to allocate preventive resources more effectively by identifying and prioritizing high-risk individuals.
- Patients, when appropriately supported, may engage with their risk profiles to guide lifestyle changes and improve cardiovascular outcomes.

These findings demonstrate the tool’s potential to enhance early CHD detection and intervention across clinical, operational, and population health contexts. Future studies should focus on real-world validation, user adoption, and longitudinal outcomes to ensure sustained clinical impact and equity in deployment.

## Supplementary Information

Supplementary Material 1.

Supplementary Material 2.

## Data Availability

The datasets were obtained from HealthInfoNet (HIN). The Maine state Health Information Exchange organization HIN is response for all data governance. All data used in this publication can be requested and access requests should be made through HIN organization CEO Shaun Alfreds salfreds@hinfonet.org.

## Abbreviations

CHD: Coronary heart disease
FRS: Framingham Risk Score
ACC/AHA13: The American College of Cardiology/American Heart Association 2013 Risk Score
AUC: Area under the curve
ML: Machine learning. EHR: Electronic health record
HIE: Health Information Exchange
ICD-10-CM: The International Classification of Disease 10th Revision, Clinical Modification
FDR: False discovery rate. Lasso: Least Absolute Shrinkage and Selection Operator. FNN: Feed-forward Neural Network
RF: Random Forest
XGBoost: Extreme Gradient Boosting
NB: Naïve Bayes
KNN: K-Nearest Neighbor
PPV: Positive predictive value
ROC: The receiver operating characteristic
CI: Confidence interval
CVD: Cardiovascular disease
OR: Odds ratio
SDOH: Social determinants of health
HIN: Health Information Network
ACO: Accountable care organization
