# Supplementary material for: Real-world risk stratification for coronary heart disease: a one-year prediction model using health information exchange data
Source: BMC Public Health. 2025 Sep 30;25:3218. doi: 10.1186/s12889-025-24266-y (PMC12486789; doi:10.1186/s12889-025-24266-y)
Supplement: Supplementary file 1 — Supplementary Material 1. [file 12889_2025_24266_MOESM1_ESM.docx]

**Real-world risk stratification for coronary heart disease: a one-year prediction model using Health Information Exchange data**

**Additional file 1**: Model technical aspects.

Table of Contents

[1 Clarification on the Relationship Between Main Text and Supplement 2](#_Toc202800140)

[2 Reporting of Confidence Intervals 2](#_Toc202800141)

[3 Feature Selection Pipeline and Justification 3](#_Toc202800142)

[4 Data Leakage Avoidance and Feature Selection Workflow 4](#_Toc202800143)

[5 Model Calibration Methodology 4](#_Toc202800144)

[6 Model Comparison and Performance Interpretation 5](#_Toc202800145)

[7 Rationale for Selecting XGBoost over LASSO as the Final Model 6](#_Toc202800146)

[8 Time-to-Event Analysis Using Cox Regression 7](#_Toc202800147)

[9 Risk Stratification and Group Definition 7](#_Toc202800148)

[10 Uncertainty Quantification and Interpretation of Risk Stratification Metrics 8](#_Toc202800149)

[11 Final Model Validation Procedures and Metrics 9](#_Toc202800150)

[12 Imputation strategy 10](#_Toc202800151)

[13 Correlation networks 11](#_Toc202800152)

# ****Clarification on the Relationship Between Main Text and Supplement****

In response to reviewer feedback, we have **elevated key methodological components** to the main text to ensure the paper is self-contained and accessible. Specifically, the revised **Methods** section in the main manuscript now includes:

- **Cohort definition**: Timeframes and population sizes for both the retrospective (training/calibration) and prospective (validation) cohorts, including criteria for CHD event labeling based on ICD-10-CM definitions.
- **Feature types**: Overview of 1,928 candidate predictors derived from longitudinal EHR data, including demographics, chronic and acute diagnoses, laboratory test abnormalities, prescription drug use, procedural codes, and healthcare utilization metrics.
- **Model development**: Description of the feature selection and model training pipeline, including statistical pre-screening, XGBoost-based importance scoring, K-fold cross-validation, and final model calibration using isotonic regression.
- **Validation approach**: Explanation of performance evaluation on the independent prospective cohort, including AUC calculation, calibration plotting, and Cox regression-based survival analysis.

The **supplementary material** continues to provide **granular methodological details**, such as:

- Justification for chi-square pre-filtering
- Encoding strategies for categorical predictors
- Imputation logic for missing laboratory values
- Hyperparameter ranges and search strategy
- Construction of correlation networks and feature interpretability via SHAP

This restructuring ensures that the main text remains **methodologically informative**, while the supplementary file provides the **technical reproducibility details** appropriate for readers seeking implementation-specific guidance.

# ****Reporting of Confidence Intervals****

To address reviewer concerns and ensure statistical rigor, we included **95% confidence intervals (CIs)** for all relevant evaluation metrics. The estimation methods are as follows:

- **AUCs**: CIs were calculated using **bootstrap resampling** (1,000 iterations) with replacement. This approach is robust to non-normality and suitable for large EHR datasets.
- **PPVs and event rates**: CIs for predicted event rates in each risk category were derived using the **Wilson score interval**, which provides accurate bounds even with small sample sizes or rare events (e.g., very high-risk group).
- **Hazard Ratios (HRs)**: Derived from **Cox proportional hazards models** fitted on the prospective cohort, with **robust standard error estimates** used to construct 95% CIs.
- **Calibration curves**: Bin-wise 95% CIs were plotted using Wilson intervals around observed incidence rates, providing a visual representation of the calibration error range across predicted risk scores (Supplementary Figure 3).
- **Survival analyses**: Kaplan–Meier survival curves include shaded 95% confidence bands for each risk group (Figure 6), enhancing interpretation of time-to-event distributions.

This consistent application of CIs ensures transparency, supports interpretation of subgroup variability, and reinforces the robustness of our model validation framework.

# ****Feature Selection Pipeline and Justification****

To address the dual challenges of extreme feature dimensionality and sparsity in statewide EHR datasets, we implemented a two-stage feature selection process designed to balance statistical rigor, predictive utility, and computational feasibility.

**Stage 1: Statistical Prescreening via Chi-square Filtering**
EHR data typically contain tens of thousands of binary and categorical variables derived from diagnosis codes, medication orders, lab flags, and procedure records. To manage this high-dimensional space, we performed chi-square testing as a prescreening step to eliminate variables with no statistically significant association with incident CHD outcomes. This unsupervised filtering reduces the burden of modeling sparse and weakly informative features while preserving potentially relevant variables without requiring parametric assumptions.

Chi-square testing is advantageous in this context because:

- It is well-suited to binary variables common in EHR datasets (e.g., presence/absence of diagnoses or lab abnormalities).
- It accommodates sparsity, since it evaluates contingency table associations rather than continuous variable distributions.

It avoids imposing prior assumptions about variable importance or interactions, thereby preventing literature-driven bias and preserving discovery of novel predictors.

**Stage 2: Predictive Feature Selection via XGBoost Variable Importance**
The reduced feature set (n = 1,928) from Stage 1 was then passed into an XGBoost classifier, which assessed the predictive contribution of each variable to the outcome. We retained 387 features with positive importance scores, reflecting their utility in improving model performance during training. This method captures nonlinear dependencies, complex interactions, and conditional relationships among features—limitations that are not addressed by the chi-square test alone.

Importantly, XGBoost's embedded feature selection capability provides robustness against overfitting and allows for ranking predictors by contribution, enabling both interpretability (via SHAP analysis) and optimization of model sparsity.

**Rationale for Combined Strategy**
This two-tiered selection framework was necessary for the following reasons:

- It reduces the risk of overfitting by excluding non-informative variables early.
- It enables scaling to statewide population-level EHR data (over 20,000 raw variables).
- It preserves interpretability through downstream SHAP-based ranking of retained variables.
- It maintains alignment with real-world EHR feature structures (sparse, binary, and imbalanced).

We emphasize that only the XGBoost-selected features were used in final model development, calibration, and validation. Thus, predictive performance is grounded in multivariate model evaluation, not in the univariate chi-square test.

# ****Data Leakage Avoidance and Feature Selection Workflow****

To prevent information leakage and ensure unbiased model evaluation, the retrospective dataset was divided into two distinct subsets before any modeling operations: (1) a **training set** for feature selection, model training, and hyperparameter tuning, and (2) a **calibration set** for model calibration via isotonic regression.

The entire feature selection process—including chi-square prescreening and tree-based variable importance scoring (XGBoost)—was confined to the training set. Importantly, no information from the calibration or prospective validation cohorts was used in the selection of predictive features. This design aligns with best practices outlined in Elements of Statistical Learning (Section 7.10.2) and avoids data leakage that could otherwise bias internal performance estimates.

**Detailed Workflow:**

- **Step 1: Data split** — The retrospective cohort was randomly split into 70% training and 30% calibration subsets.
- **Step 2: Feature prescreening** — Within the training set, we removed variables with high missingness or extreme imbalance, followed by chi-square filtering to identify features associated with CHD outcomes.
- **Step 3: Predictive feature selection** — The resulting candidate features were input into an XGBoost model, and features with positive importance scores were retained (n = 387).
- **Step 4: Hyperparameter tuning** — A grid search combined with K-fold cross-validation (within the training set) was used to identify optimal model settings.
- **Step 5: Calibration** — The calibration set was used only to map predicted risk scores to positive predictive values using isotonic regression.
- **Step 6: Prospective validation** — An independent prospective cohort was used for final model evaluation.

This pipeline ensures that feature selection and model evaluation are conducted on disjoint datasets, thereby mitigating the risk of overfitting or overestimating model performance. Although nested cross-validation is ideal in smaller datasets, our approach reflects a compromise between statistical rigor and computational tractability in a real-world, population-scale EHR setting.

# ****Model Calibration Methodology****

Accurate calibration ensures that predicted probabilities reflect the true risk of outcomes across the entire prediction spectrum, not just the average. To address this, we employed **isotonic regression** to calibrate our CHD risk prediction model.

**Rationale for Isotonic Regression:**

- Isotonic regression is a **nonparametric** method that fits a monotonic mapping between uncalibrated model outputs and observed event rates.
- Unlike parametric methods (e.g., Platt scaling), isotonic regression **does not assume a specific functional form** (such as a sigmoid curve), making it better suited for **heterogeneous real-world EHR data** where score-to-risk relationships may be irregular.
- It provides **local adaptivity**, especially useful in skewed or imbalanced datasets, allowing for more accurate mapping in regions with sparse data or nonlinear trends.

**Implementation Steps:**

1. After model training on the retrospective training set, predicted risk scores were generated for the **hold-out calibration subset**.
2. The calibration mapping was learned using **isotonic regression**, with predicted scores as inputs and the actual 1-year CHD outcome labels as targets.
3. The fitted isotonic model was then used to **map raw prediction scores to calibrated probabilities** (i.e., estimated positive predictive values, PPVs).
4. Calibrated risk probabilities were then applied to the independent prospective cohort for validation and stratification into clinical risk categories.

Our results confirm that the isotonic regression mapping maintained good calibration across all risk levels, including both high and low predicted probabilities.

We selected isotonic regression over Platt scaling due to its **greater flexibility, superior empirical performance in large-scale clinical datasets**, and ability to preserve monotonicity without risking parametric misspecification.

# ****Model Comparison and Performance Interpretation****

To benchmark our predictive framework, we systematically evaluated multiple machine learning algorithms: LASSO, XGBoost, Random Forest, Feed-forward Neural Network (FNN), Naïve Bayes, k-Nearest Neighbor (KNN), and an ensemble classifier that aggregated their predictions.

**Observations:**

- Ensemble and XGBoost models outperformed others, achieving AUCs of 0.888 and 0.871, respectively.
- LASSO achieved comparable AUCs to XGBoost, despite being a linear model.
- Random Forest and FNN underperformed relative to expectation, given the large dataset and moderate feature dimensionality.

**Possible Explanations:**

1. **Data modality and sparsity**
   Our EHR dataset comprises >1,000 binary/categorical variables with high missingness and low marginal frequencies. This favors algorithms like LASSO and XGBoost, which are optimized for tabular, sparse data. In contrast, Random Forests and FNNs—although theoretically powerful—require dense and well-structured feature representations to realize their potential. Without feature embeddings or engineered transformations, their performance is diminished.
2. **Noise and regularization mismatch**
   Despite the large sample size, the underlying signal-to-noise ratio is modest due to inherent heterogeneity in clinical data and outcome labeling uncertainty. FNNs and Random Forests, with high flexibility, are more susceptible to overfitting noisy or redundant patterns unless heavily regularized. XGBoost's built-in regularization (L1 and L2), pruning, and shrinkage mitigate this risk, enabling it to extract weak signals across many weakly informative features.
3. **Hyperparameter tuning scope**
   While we employed grid search for key hyperparameters (e.g., tree depth, learning rate, number of trees for XGBoost; number of neurons, epochs, and dropout rate for FNN), resource constraints limited deeper nested optimization. It is plausible that wider or adaptive search strategies (e.g., Bayesian optimization) might improve the performance of neural and ensemble tree-based models, though likely with diminishing returns in this tabular context.
4. **Feature encoding and architecture compatibility**
   LASSO and XGBoost exploit the high sparsity of binary/categorical data effectively. Neural networks, in contrast, are often hindered by such sparsity unless enhanced with embeddings or domain-specific architectural adjustments. Given our model goal of scalable deployment on statewide data, we prioritized parsimonious, interpretable architectures over deep or recurrent networks.

**Conclusion:**
These results highlight the importance of aligning modeling choice with data characteristics. While complex learners offer greater flexibility, they do not universally outperform simpler models, especially in structured EHR domains where regularized, tree-based methods like XGBoost can balance interpretability, performance, and scalability.

# ****Rationale for Selecting XGBoost over LASSO as the Final Model****

Both LASSO and XGBoost were strong candidates for final model selection, having demonstrated comparable discrimination performance (AUC) in the prospective cohort. However, several technical and practical considerations led us to select XGBoost as the final model for deployment:

1. **Modeling Non-Linearity and Interactions**
   - LASSO models linear associations between predictors and outcomes. This limits its ability to model non-linear relationships, interaction effects, and threshold-based decision logic—common in clinical data.
   - XGBoost, a gradient-boosted decision tree method, captures complex, non-linear interactions between features without requiring manual interaction terms or basis expansions.
2. **Data Robustness and Missingness**
   - Real-world EHR data are noisy, sparse, and contain many missing values. LASSO requires explicit imputation, which may introduce bias.
   - XGBoost handles missing values natively during training and prediction, using learned default directions within tree nodes. This makes it more robust in the presence of incomplete records and minimizes preprocessing burden.
3. **Feature Interpretability and Clinical Transparency**
   - LASSO’s coefficient shrinkage provides a transparent view of selected features but does not explain individual predictions.
   - XGBoost integrates seamlessly with SHAP, allowing detailed attribution of prediction risk to specific features for each individual—facilitating interpretability in clinical dashboards and supporting model auditability.
4. **Scalability and Maintainability**
   - XGBoost’s scalability allows rapid retraining and model updates in large-scale systems like Health Information Exchanges (HIEs).
   - Its built-in regularization (L1 and L2) ensures stability without sacrificing flexibility, especially when adapting to evolving feature distributions or incorporating new data sources.
5. **Deployment Alignment**
   - XGBoost’s prediction speed, cross-platform support (e.g., Python, R, Java), and compatibility with cloud infrastructure make it ideal for production deployment in statewide health systems.

In conclusion, while LASSO demonstrated respectable performance and simplicity, XGBoost provided superior adaptability, robustness, and interpretability—key considerations for translating predictive analytics into actionable, scalable, and trusted clinical tools.

# ****Time-to-Event Analysis Using Cox Regression****

To evaluate the predictive validity of our CHD risk model beyond classification metrics (e.g., AUC), we incorporated **time-to-event analyses** using **Cox proportional hazards models** within the **prospective cohort**, which served as an **independent validation set**.

**Process Overview:**

- After model training and optimization on the retrospective cohort (as outlined in Figure 2), the finalized XGBoost model was applied to the prospective cohort (N = 1,040,158).
- Each individual in the prospective cohort received a predicted risk score, which was then **calibrated using isotonic regression** and mapped to one of five predefined risk categories (very low, low, medium, high, very high).
- Using these risk strata, **Cox regression models** were fitted to assess the relative hazard of developing incident CHD during the one-year follow-up period.
- **Kaplan–Meier curves** and **hazard ratios (HRs)** were generated to quantify survival differences across groups. These are displayed in Figure 6 of the main manuscript.

**Justification:**
This approach ensures that the time-to-event evaluation is conducted on completely unseen data, preserving the integrity of validation and avoiding optimistic bias. The Cox model complements the ROC-based evaluation by confirming that risk stratification corresponds to significantly different CHD incidence trajectories.

**Software and Implementation:**
Cox regression and Kaplan–Meier analyses were implemented in **R (v4.2)** using the survival and survminer packages. Confidence intervals for HRs were derived using robust standard error estimates.

This validation strategy supports the model’s utility for **both binary classification** and **longitudinal risk stratification**, reinforcing its translational potential in clinical and public health settings.

# ****Risk Stratification and Group Definition****

The predictive output of the final model—a calibrated probability y^∈[0,1]\hat{y}\in [0,1]y^∈[0,1]—was used to assign each patient in the **prospective validation cohort** to one of five **ordinal risk categories** reflecting their estimated 1-year CHD risk.

**Calibration-Based Risk Scoring**
Following application of the XGBoost model to the prospective dataset, each patient’s raw model score was passed through an **isotonic regression** mapping derived from the retrospective cohort calibration subset. This mapping converted the raw prediction to an empirically observed **positive predictive value (PPV)**, corresponding to the **estimated incidence rate** of CHD over the next year.

**Risk Group Cutoff Determination**
The calibrated PPVs were used to divide the population into five strata:

- **Very low risk**: individuals with predicted risk scores in the lowest range (covering ~92% of the population)
- **Low risk**: next tier of risk (covering ~6.8%)
- **Medium risk**: intermediate scores (~0.85%)
- **High risk**: upper range (~0.05%)
- **Very high risk**: top 0.002%, corresponding to the highest predicted risk group

These breakpoints were chosen to balance **clinical interpretability**, **statistical robustness**, and **practical utility**, ensuring sufficient sample size within each group to allow meaningful subgroup analysis (e.g., survival curves, feature distributions).

**Justification for Using PPV as Risk Metric**
PPV was chosen over raw model output or deciles because it reflects the **true event rate** conditional on the risk score. This enables direct interpretation of model output in terms of likelihood of incident CHD, aligning with real-world decision-making for preventive interventions. The use of calibrated PPVs as thresholds improves **comparability across populations** and supports deployment within health information systems.

**Visualization and Outcome Validation**
The distribution of predicted scores and CHD incidence across the five risk strata is visualized in **Figure 5**, and time-to-event outcomes are validated via **Kaplan–Meier survival curves** in **Figure 6**. These figures confirm the ordinal validity of risk groups and their discriminative capacity.

# ****Uncertainty Quantification and Interpretation of Risk Stratification Metrics****

To ensure transparent and statistically sound interpretation of the CHD prediction model’s performance, we incorporated **95% confidence intervals (CIs)** for all key evaluation metrics:

- **Model Discrimination**: AUCs were estimated with 95% CIs using bootstrap resampling (1,000 iterations).
- **Model Calibration**: Observed vs. predicted risks were plotted with bin-wise 95% CIs, using Wilson score intervals.
- **Survival Analysis**: Kaplan–Meier curves include 95% CIs for each risk group; Cox hazard ratios were reported with robust standard error-based confidence intervals.

These measures are presented in the main text (Figure 3 and Figure 6) and supplementary materials (Supplementary Figure 3 for calibration, and Supplementary Table 2 for stratified performance).

**Note on High and Very-High Risk Group Uncertainty:**

- The **high (n=554)** and **very high (n=19)** risk categories comprise a small proportion of the prospective cohort.
- Due to limited sample sizes, particularly in the very high-risk stratum, statistical volatility is **expected and acknowledged**. These strata are nonetheless retained to support **clinical prioritization**, recognizing the exploratory nature of their estimates.
- The wide confidence intervals in these groups should be interpreted with caution. As noted in the main text, future prospective cohort expansion will be needed to **improve the precision** of calibration and performance estimates in these upper-risk tiers.

**Clarification on Relative Risk (RR) Values:**

- The **mean relative risk** values in Supplementary Table 2 are derived as the **ratio of subgroup-specific CHD incidence** to the **overall population incidence rate** (i.e., RR = PPV_group / PPV_overall).
- These values are calculated deterministically from known prevalence figures and therefore do **not represent sampling-based estimates**. As such, confidence intervals are not provided for RR values.

This methodological transparency supports responsible interpretation of model performance, particularly in rare-event subgroups where caution is warranted.

# ****Final Model Validation Procedures and Metrics****

To rigorously assess the performance of the final CHD risk prediction model (XGBoost), we conducted both **discrimination** and **calibration** evaluations on an **independent, prospective validation cohort (N=1,040,158)**.

**1. Discrimination Performance**

- Receiver operating characteristic (ROC) curves were generated for both training (retrospective) and validation (prospective) datasets.
- Area under the curve (AUC) metrics were reported with **95% confidence intervals (CIs)** using bootstrapping with 1,000 resamples.
  - Retrospective AUC: 0.952 (CI: 0.950–0.954)
  - Prospective AUC: 0.888 (CI: 0.885–0.890)
- These results demonstrate strong model discrimination and generalizability across cohorts.

**2. Calibration Assessment**

- Model calibration was evaluated by comparing observed CHD incidence rates with predicted probabilities (PPVs) across score bins.
- Calibration curves were generated with 95% bin-wise CIs using Wilson score intervals (Supplementary Figure 3).
- Calibration accuracy was further summarized by stratifying individuals into five ordinal risk categories (very low to very high) and comparing predicted PPVs with actual incidence rates (Supplementary Table 2).

**3. Time-to-Event Validation**

- Individuals in the prospective cohort were grouped based on calibrated predicted risk.
- Cox proportional hazards regression was used to estimate hazard ratios (HRs) for CHD incidence across strata.
- Kaplan–Meier curves and HRs (Figure 6) validated progressive risk separation, with HRs ranging from 9.27 to 97.48 across groups.

**4. Clinical Implication of 1-Year Prediction Window**

- The 1-year prediction interval corresponds to a critical window for **intervention prior to plaque rupture**, with evidence suggesting average transition time from vulnerable to unstable plaque is approximately 12 months.
- This timeframe allows actionable interventions (lipid-lowering, metabolic modulation, anti-inflammatory therapy) for **early-stage subclinical atherosclerosis**, making the model clinically actionable for care planning and health system resource allocation.

Together, these validation strategies support the model’s reliability and clinical utility across both individual- and population-level decision-making.

# Imputation Strategy

In primary care settings, particularly for cardiovascular risk assessment, clinicians selectively order tests based on clinical suspicion. The absence of a particular test in a patient's record typically indicates one of two scenarios: (1) the clinician did not consider the test clinically necessary based on the patient's presentation, suggesting a presumed normal status; or (2) the test was truly missing due to administrative or documentation errors.

While scenario (2) represents true missing data, our clinical collaborators confirmed that scenario (1) is substantially more common in routine practice. Therefore, imputing missing values as 0 ("normal") aligns with the clinical decision-making process that generated our data. Alternative approaches like mean imputation or multiple imputation would likely introduce greater bias by ignoring this clinical context.

We acknowledge the limitations of this approach. To validate our methodology, we performed sensitivity analyses using alternative imputation strategies on a subset of data where we could confirm the true reason for missing tests. The zero-imputation approach demonstrated superior predictive performance and better clinical face validity compared to alternatives.

This imputation strategy represents a balance between statistical rigor and clinical reality in working with real-world EHR data. Future work will explore more sophisticated imputation methods that incorporate clinical context and temporal patterns to further refine our approach.

# Correlation Networks

Correlation networks were constructed using the selected features by performing Spearman's correlations on the study subjects. Within these networks, the vertices corresponded to the features, and any edge that existed between any pair of vertices represented a significant correlation (with an absolute value of the Spearman coefficient exceeding 0.1) between each pair of features. In actual clinical settings, these significant features are unlikely to be independent of each other, and more complicated causative and/or associative relationships might exist among these features.
