# Supplementary material for: Real-world risk stratification for coronary heart disease: a one-year prediction model using health information exchange data
Source: BMC Public Health. 2025 Sep 30;25:3218. doi: 10.1186/s12889-025-24266-y (PMC12486789; doi:10.1186/s12889-025-24266-y)

**Real-world risk stratification for coronary heart disease: a one-year prediction model using Health Information Exchange data**

**Additional file 2:** Supplementary Tables and Figures

Table of Contents

[1 Supplementary Table 1. 2](#_Toc202796121)

[2 Supplementary Table 2. 4](#_Toc202796122)

[3 Supplementary Figure 1. 4](#_Toc202796123)

[4 Supplementary Figure 2. 5](#_Toc202796124)

[5 Supplementary Figure 3. 6](#_Toc202796125)

[6 Supplementary Figure 4. 7](#_Toc202796126)

[7 Supplementary Figure 5. 8](#_Toc202796127)

[8 Supplementary Figure 6. 8](#_Toc202796128)

# Supplementary Table 1.

List of the top 80 important features, their odds ratios, and importances in the model.

| **Age(years)** | **Prospective cohort** | **Case ^a^ (N=15,781)** | **n%** | **Control ^b^ (N=1,024,391)** | **n%** | **Odds ratio** | **Importance**  **(×10^-3^)** |
| --- | --- | --- | --- | --- | --- | --- | --- |
| <19 | 215,842 | 21 | 0.13 | 215,821 | 21.07 | 0.02 | 64.61 |
| 19-34 | 186,586 | 162 | 1.03 | 186,424 | 18.20 | 0.05 | 59.67 |
| 35-49 | 178,568 | 854 | 5.41 | 177,714 | 17.35 | 0.27 | 33.45 |
| 50-64 | 240,285 | 4,328 | 27.43 | 235,957 | 23.03 | 1.26 | 13.27 |
| 65-74 | 132,265 | 5,035 | 31.91 | 127,230 | 12.42 | 3.30 | 35.20 |
| 75-84 | 61,150 | 3,710 | 23.51 | 57,440 | 5.61 | 5.17 | 42.66 |
| >85 | 25,476 | 1,671 | 10.59 | 23,805 | 2.32 | 4.98 | 48.51 |
| **Gender** |  |  |  |  |  |  |  |
| Male | 461,013 | 8,897 | 56.38 | 452,116 | 44.14 | 1.64 | 13.8 |
| Female | 579,159 | 6,884 | 43.62 | 572,275 | 55.86 | 1.64 | 13.8 |
| **Chronic disease** |  |  |  |  |  |  |  |
| Heart failure (CVD) | 12,961 | 1,528 | 9.68 | 11,433 | 1.12 | 9.50 | 4.07 |
| Atrial fibrillation and flutter (CVD) | 27,418 | 2,305 | 14.61 | 25,113 | 2.45 | 6.81 | 3.59 |
| Cardiomyopathy (CVD) | 5,243 | 601 | 3.81 | 4,642 | 0.45 | 6.70 | 2.14 |
| Nonrheumatic aortic valve disorders (CVD) | 9,356 | 901 | 5.71 | 8,455 | 0.83 | 7.28 | 1.7 |
| Paroxysmal tachycardia (CVD) | 7,381 | 400 | 2.53 | 6,981 | 0.68 | 3.79 | 1.1 |
| Complications and ill-defined descriptions of heart disease (CVD) | 10,886 | 947 | 6.00 | 9,939 | 0.97 | 6.52 | 0.63 |
| Atrioventricular and left bundle-branch block (CVD) | 5,018 | 472 | 2.99 | 4,546 | 0.44 | 6.93 | 0.4 |
| Nonrheumatic mitral valve disorders (CVD) | 11,397 | 837 | 5.30 | 10,560 | 1.03 | 5.38 | 0.21 |
| Chronic obstructive pulmonary disease | 38,390 | 2,618 | 16.59 | 35,772 | 3.49 | 5.50 | 6.47 |
| Occlusion and stenosis of precerebral artery, not result in cerebral infarction | 6,701 | 708 | 4.49 | 5,993 | 0.59 | 7.98 | 2.04 |
| Chronic kidney disease | 21,228 | 1,728 | 10.95 | 19,500 | 1.90 | 6.34 | 1.6 |
| Angina pectoris | 1,620 | 221 | 1.40 | 1,399 | 0.14 | 10.39 | 1.49 |
| Other peripheral vascular diseases | 11,853 | 936 | 5.93 | 10,917 | 1.07 | 5.85 | 1.41 |
| Overweight and obesity | 85,694 | 2,461 | 15.59 | 83,233 | 8.13 | 2.09 | 1.21 |
| Hypothyroidism | 74,878 | 2,298 | 14.56 | 72,580 | 7.09 | 2.23 | 0.91 |
| Aortic aneurysm and dissection | 4,934 | 452 | 2.86 | 4,482 | 0.44 | 6.71 | 0.66 |
| **Acute disease** |  |  |  |  |  |  |  |
| Acute myocardial infarction | 1,821 | 437 | 2.77 | 1,384 | 0.14 | 21.05 | 4.7 |
| Pain in throat and chest | 52,346 | 1,758 | 11.14 | 50,588 | 4.94 | 2.41 | 2.49 |
| Abnormalities of breathing | 46,075 | 2,022 | 12.81 | 44,053 | 4.30 | 3.27 | 1.62 |
| Other soft tissue diseases | 82,254 | 2,295 | 14.54 | 79,959 | 7.81 | 2.01 | 1.09 |
| Abnormal findings on diagnostic imaging of lung | 12,909 | 775 | 4.91 | 12,134 | 1.18 | 4.31 | 0.83 |
| Other functional intestinal disorders | 24,985 | 682 | 4.32 | 24,303 | 2.37 | 1.86 | 0.67 |
| Elevated blood glucose level | 27,916 | 805 | 5.10 | 27,111 | 2.65 | 1.98 | 0.66 |
| Cough | 56,272 | 1,430 | 9.06 | 54,842 | 5.35 | 1.76 | 0.62 |
| Abnormalities of heart beat | 26,145 | 977 | 6.19 | 25,168 | 2.46 | 2.62 | 0.61 |
| **Disease events** |  |  |  |  |  |  |  |
| Essential (primary) hypertension | 56,678 | 2,235 | 14.16 | 54,443 | 5.31 | 2.76 | 3.37 |
| Type 2 diabetes mellitus without complications | 29,296 | 1,467 | 9.30 | 27,829 | 2.72 | 2.76 | 3.57 |
| Chronic obstructive pulmonary disease | 10,682 | 856 | 5.42 | 9,826 | 0.96 | 2.92 | 0.95 |
| Hyperlipidemia | 647 | 48 | 0.30 | 599 | 0.06 | 5.94 | 1.08 |
| **Health status** |  |  |  |  |  |  |  |
| Presence of cardiac and vascular implants and grafts | 6,010 | 742 | 4.70 | 5,268 | 0.51 | 1.19 | 5.86 |
| Tobacco use | 19,356 | 505 | 3.20 | 18,851 | 1.84 | 1.30 | 1.54 |
| Body mass index>30 | 59,753 | 1,775 | 11.25 | 57,978 | 5.66 | 1.15 | 1.7 |
| Personal history of certain other diseases | 39,993 | 1,791 | 11.35 | 38,202 | 3.73 | 1.20 | 1.27 |
| Encounter for other aftercare and medical care | 16,139 | 761 | 4.82 | 15,378 | 1.50 | 1.08 | 1.11 |
| Do not resuscitate | 6,034 | 309 | 1.96 | 5,725 | 0.56 | 1.64 | 0.92 |
| Other postprocedural states | 31,456 | 1,041 | 6.60 | 30,415 | 2.97 | 1.20 | 0.88 |
| Personal history of malignant neoplasm | 30,741 | 1,295 | 8.21 | 29,446 | 2.87 | 1.21 | 0.82 |
| Presence of other functional implants | 21,607 | 993 | 6.29 | 20,614 | 2.01 | 1.18 | 0.77 |
| **Lab test** |  |  |  |  |  |  |  |
| Erythrocyte distribution width RBC | 61,238 | 2,089 | 13.24 | 59,149 | 5.77 | 1.06 | 1.76 |
| Platelets in Blood | 73,342 | 2,274 | 14.41 | 71,068 | 6.94 | 1.06 | 1.5 |
| Basophils in Blood | 63,453 | 1,801 | 11.41 | 61,652 | 6.02 | 1.09 | 1.48 |
| Cholesterol in high-density lipoprotein | 29,979 | 961 | 6.09 | 29,018 | 2.83 | 1.83 | 1.36 |
| Anion gap in Serum or Plasma | 58,451 | 2,000 | 12.67 | 56,451 | 5.51 | 1.07 | 1.29 |
| Hemoglobin A1c/Hemoglobin.total in Blood | 19,086 | 1,069 | 6.77 | 18,017 | 1.76 | 2.02 | 1.26 |
| Glomerular filtration rate predicted among non-blacks | 17,453 | 891 | 5.65 | 16,562 | 1.62 | 1.08 | 1.11 |
| Magnesium in Serum or Plasma | 21,903 | 1,051 | 6.66 | 20,852 | 2.04 | 1.12 | 1.09 |
| Triglyceride in Serum or Plasma | 35,821 | 1,099 | 6.96 | 34,722 | 3.39 | 1.49 | 0.99 |
| Eosinophils in Blood | 68,390 | 2,002 | 12.69 | 66,388 | 6.48 | 1.09 | 0.96 |
| Glomerular filtration rate predicted among blacks | 33,897 | 1,339 | 8.48 | 32,558 | 3.18 | 1.08 | 0.95 |
| Cholesterol in Serum or Plasma | 44,206 | 1,309 | 8.29 | 42,897 | 4.19 | 1.70 | 0.89 |
| Estimated average glucose | 6,744 | 464 | 2.94 | 6,280 | 0.61 | 3.08 | 0.83 |
| Glucose in Capillary blood | 14,137 | 667 | 4.23 | 13,470 | 1.31 | 1.01 | 0.67 |
| INR in Blood by Coagulation assay | 12,865 | 864 | 5.47 | 12,001 | 1.17 | 1.09 | 0.65 |
| **Medication** |  |  |  |  |  |  |  |
| Nitrate Vasodilator | 4,197 | 997 | 6.32 | 3,200 | 0.31 | 1.28 | 42.04 |
| Loop Diuretic | 18,799 | 1,911 | 12.11 | 16,888 | 1.65 | 1.10 | 25.79 |
| P2Y12 Platelet Inhibitor | 2,980 | 470 | 2.98 | 2,510 | 0.25 | 1.14 | 22.46 |
| Dihydropyridine Calcium Channel Blocker | 27,093 | 1,861 | 11.79 | 25,232 | 2.46 | 1.10 | 14.29 |
| Biguanide | 29,234 | 1,856 | 11.76 | 27,378 | 2.67 | 1.08 | 11.32 |
| Insulin Analog | 13,795 | 1,110 | 7.03 | 12,685 | 1.24 | 1.05 | 10.70 |
| Angiotensin 2 Receptor Blocker | 26,001 | 1,672 | 10.60 | 24,329 | 2.37 | 1.09 | 9.58 |
| Alpha-Adrenergic Blocker | 22,996 | 1,678 | 10.63 | 21,318 | 2.08 | 1.07 | 9.33 |
| Thiazide Diuretic | 39,616 | 1,902 | 12.05 | 37,714 | 3.68 | 1.08 | 8.36 |
| Anticholinergic | 23,007 | 1,320 | 8.36 | 21,687 | 2.12 | 1.05 | 4.82 |
| l-Thyroxine | 47,601 | 1,976 | 12.52 | 45,625 | 4.45 | 1.06 | 4.60 |
| Anti-epileptic Agent | 44,055 | 1,664 | 10.54 | 42,391 | 4.14 | 1.02 | 4.52 |
| Vitamin K Antagonist | 11,009 | 1,051 | 6.66 | 9,958 | 0.97 | 1.05 | 4.42 |
| Osmotic Laxative | 25,411 | 1,249 | 7.91 | 24,162 | 2.36 | 1.06 | 2.20 |
| Sulfonylurea | 13,524 | 1,028 | 6.51 | 12,496 | 1.22 | 1.08 | 2.12 |
| Calcium Channel Blocker | 9,246 | 706 | 4.47 | 8,540 | 0.83 | 1.09 | 1.97 |
| Antiarrhythmic | 14,160 | 764 | 4.84 | 13,396 | 1.31 | 1.09 | 1.75 |
| Factor Xa Inhibitor | 6,911 | 567 | 3.59 | 6,344 | 0.62 | 1.08 | 1.09 |
| Xanthine Oxidase Inhibitor | 6,889 | 572 | 3.62 | 6,317 | 0.62 | 1.10 | 0.93 |
| Potassium Salt | 14,850 | 968 | 6.13 | 13,882 | 1.36 | 1.09 | 0.77 |
| **Utilization** |  |  |  |  |  |  |  |
| Mean of medical cost(s) | - | 4,797.06 | - | 2,056.16 | - | 1.00 | 19.1 |
| Mean of outpatient visit(s) | - | 10.27 | - | 5.16 | - | 1.05 | 16.62 |
| Mean of emergency visit(s) | - | 0.65 | - | 0.45 | - | 1.07 | 8.81 |
| Mean of inpatient day(s) | - | 1.41 | - | 0.52 | - | 1.00 | 3.18 |
| Mean of inpatient admission(s) | - | 0.22 | - | 0.09 | - | 1.51 | 1.3 |

^a^ Case is the patients with future one year CHD.

^b^ Control is the people without future one year CHD but with other conditions similar to the case group.

# Supplementary Table 2.

The performance of the 1-year CHD risk pre-judgement model in the prospective cohort, summarized in PPV, sensitivity, and mean relative risk.

|  | **Very low** | **Low** | **Medium** | **High** | **Very high** | **Total** |
| --- | --- | --- | --- | --- | --- | --- |
| **Number of patients** | 960,021 | 70,676 | 8,888 | 554 | 19 | 1,040,158 |
| **True Positives** | 8,367 | 5,506 | 1,668 | 216 | 11 | 15,768 |
| **PPV (%)** | 0.87 | 7.79 | 18.77 | 38.99 | 57.89 | 1.6 |
| **Sensitivity (%)** | 0.53 | 0.35 | 0.11 | 0.01 | 0.00 | - |
| **Mean Relative Risk** | 0.57 | 5.14 | 12.38 | 25.72 | 38.19 | - |

# Supplementary Figure 1.

The AUC comparisons of the model performance in the prospective cohort. 1. Ensemble learning, 2. XGBoost, 3. Lasso, 4. Boosting, 5. Feed-forward neural network, 6. Random forest, 7. KNN, 8. Naïve bayes.

# Supplementary Figure 2.

The ROC derived from the prospective cohorts (XGBoost).


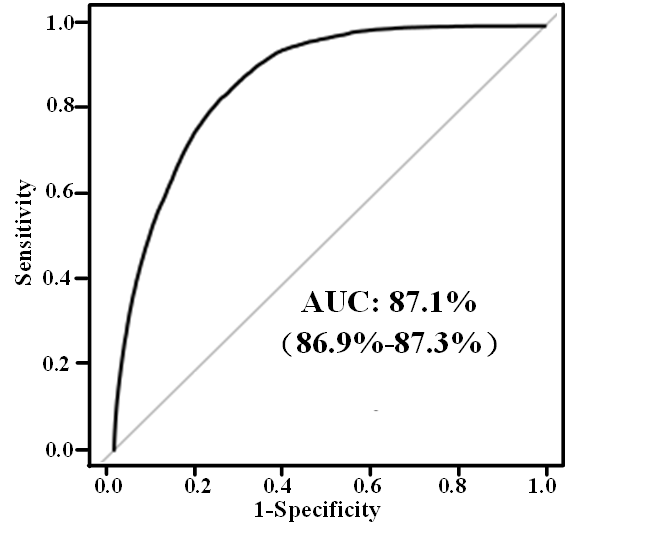


# Supplementary Figure 3.

The model calibration performance.

**
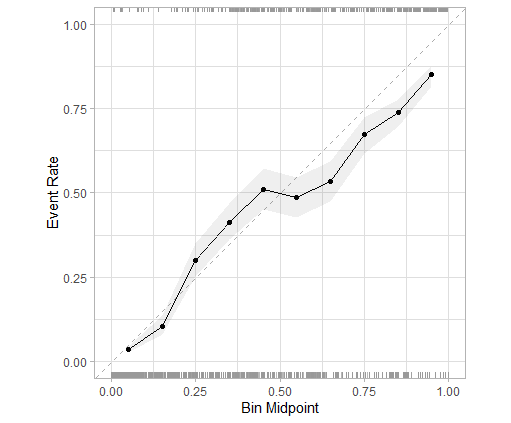
**

# Supplementary Figure 4.

The TreeSHAP values of the significant features. Each row represents a significant feature, and each point is the TreeSHAP value of a sample. Redder sample points the feature value is larger, and bluer sample points indicate that the feature value is smaller; the abscissae represent the TreeSHAP values.


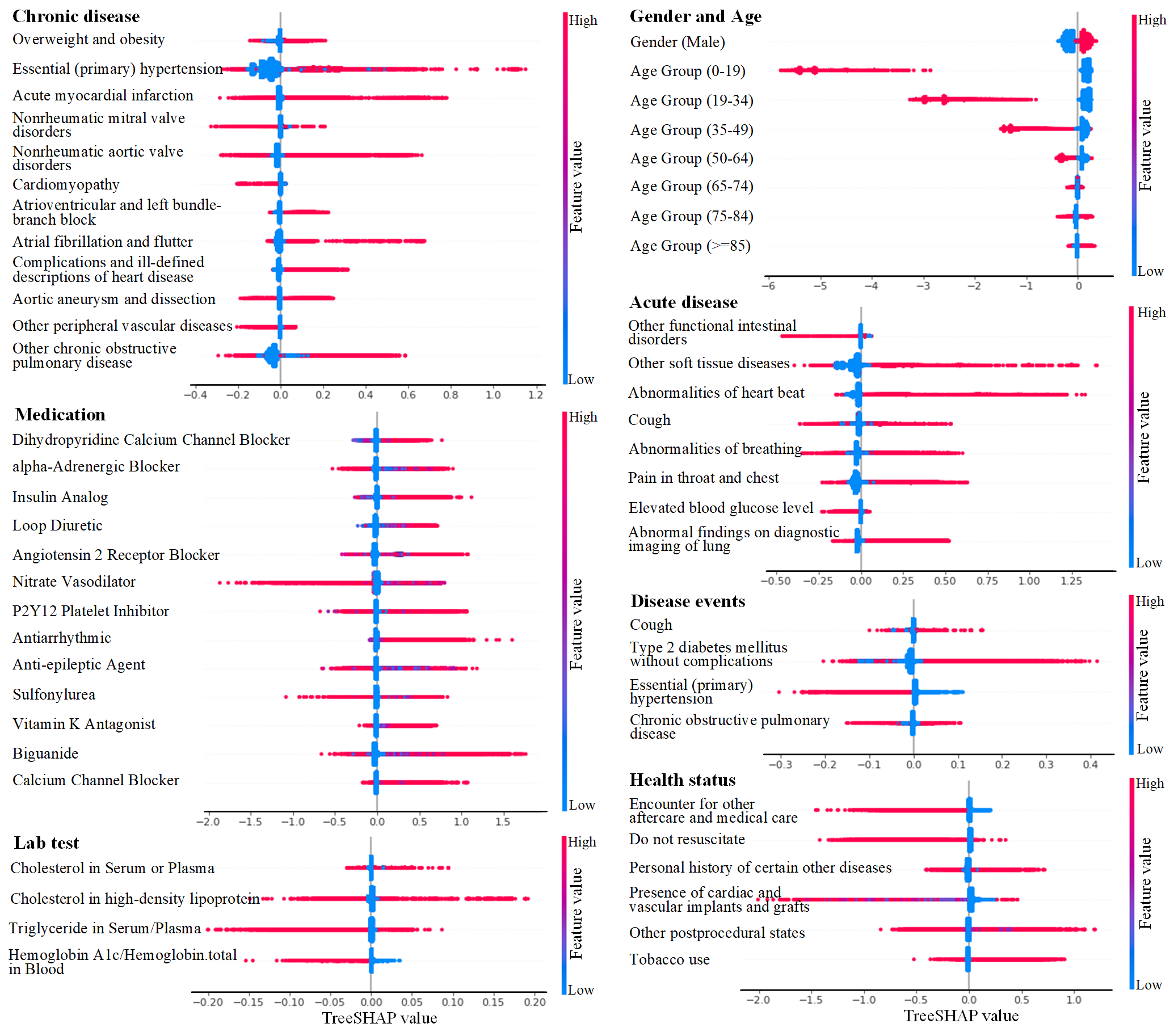


# Supplementary Figure 5.

The distribution of age and gender (male) subgroups at five risk level in the prospective cohort.


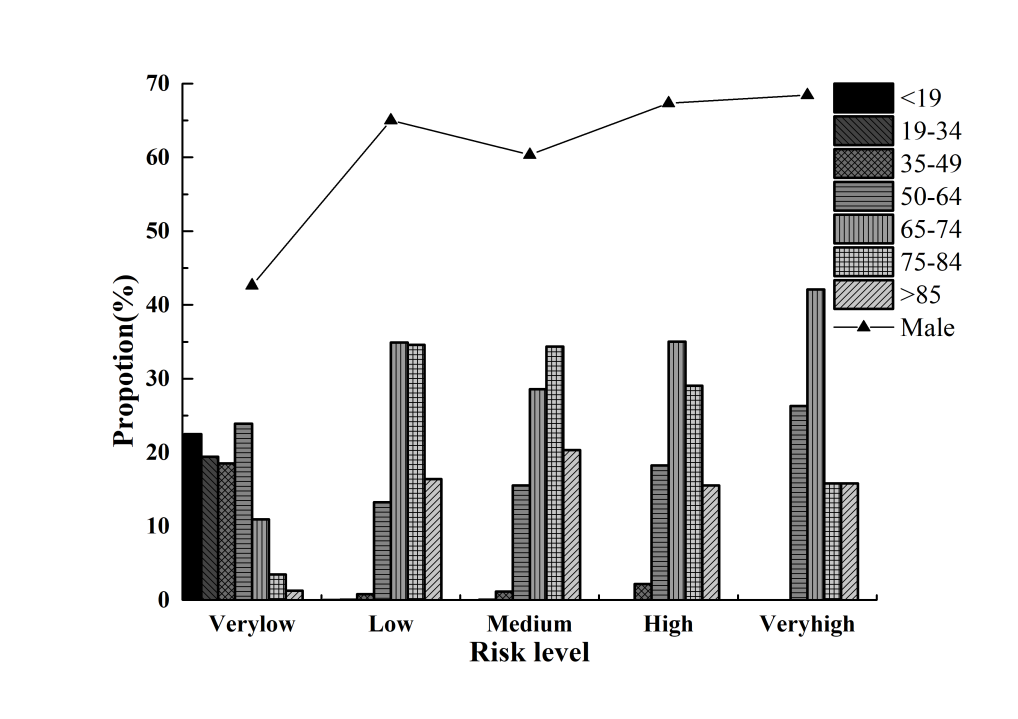


# Supplementary Figure 6.

Time-to-CHD diagnosis curves of the chronic disease subgroup in the low/ very low and high/ very high-risk population of prospective cohort.


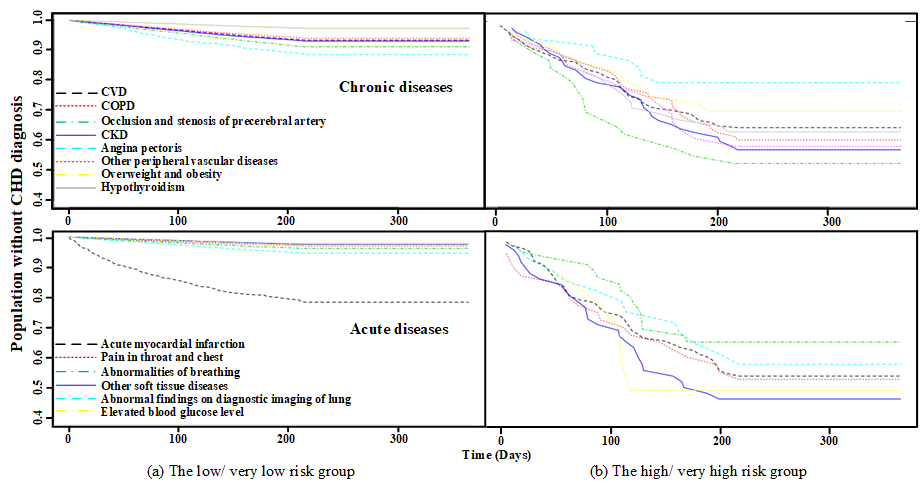

Supplement: Supplementary file 2 — Supplementary Material 2. [file 12889_2025_24266_MOESM2_ESM.docx]
